# Evaluating bromeliads as environmental reservoirs for *Batrachochytrium dendrobatidis* and amphibian disease dynamics in equatorial forests

**DOI:** 10.1098/rsos.252148

**Published:** 2026-09-09

**Authors:** MDM Moretta-Urdiales, Shawn McCracken, Juan M. Guayasamin, Ryan L. Lynch, Moises Tenorio, Wesley J. Neely, David Rodriguez

**Affiliations:** ^1^ Department of Biology, Texas State University, San Marcos, TX, USA; ^2^ Department of Life Sciences, Texas A&M University-Corpus Christi, Corpus Christi, TX, USA; ^3^ Laboratorio de Biología Evolutiva, Universidad San Francisco de Quito, Quito, Ecuador; ^4^ Tandayapa Cloud Forest Station, Universidad San Francisco de Quito, Quito, Ecuador; ^5^ Third Millennium Alliance, Fremont, CA, USA

**Keywords:** canopy ecology, chytrid fungus, disease ecology, Ecuador, eDNA, frogs, host-pathogen dynamics

## Abstract

The amphibian chytrid fungus *Batrachochytrium dendrobatidis* (Bd) is widely distributed across tropical ecosystems, yet the microhabitats that facilitate its environmental persistence remain poorly understood. We evaluated Bd detection in bromeliad tank contents and infection in amphibian communities across an elevational gradient in Ecuadorian forests. We sampled amphibians at eight sites using skin swabs, and canopy and subcanopy bromeliads at five sites using eDNA. Overall, Bd prevalence was 23.0% in amphibians and 11.3% in bromeliads. Subcanopy bromeliads showed a significantly lower probability of Bd detection than amphibians, whereas canopy bromeliads did not differ significantly from amphibians or subcanopy bromeliads. Mid-elevation sites showed a higher probability of being Bd-positive compared with high-elevation sites, while low-elevation sites did not differ significantly. Amphibian Bd prevalence also increased with precipitation and followed a nonlinear relationship with temperature, suggesting higher prevalence under intermediate thermal conditions. By contrast, Bd detection in bromeliads was not significantly associated with abiotic factors. Bd infection intensity among amphibians did not differ significantly across host taxonomy or sampling sites. Together, these results suggest that bromeliad tanks may provide aquatic microhabitats for Bd detection and short-term persistence, but host infection patterns appear more associated with amphibian taxonomic identity, site-level variation and local climatic conditions.

## 1. Introduction

Amphibians are currently facing an unprecedented global panzootic driven by the fungal pathogen *Batrachochytrium dendrobatidis* (Bd), which has contributed to population declines and extinction events across a wide range of ecosystems [1,2]. Although Bd is often associated with humid environments and temperatures within its commonly reported growth range, approximately 17–25°C [3–5], it has also been detected in lowland ecosystems where temperatures can reach 32°C [6,7], suggesting that broad macroclimatic conditions alone do not fully explain where the pathogen spreads. In Ecuador, Bd has been reported across all three mainland regions [8–13], including areas where environmental conditions do not seem favourable for pathogen survival. This broad distribution raises questions about the type of local habitats or microenvironmental conditions that facilitate Bd persistence in tropical forest ecosystems.

One microhabitat that may be especially relevant to this question is the tank bromeliad. Many bromeliads accumulate rainwater and organic matter between their leaves, creating aquatic microhabitats that are widely used by amphibians for shelter, reproduction and larval development [14–18]. This ecological role is particularly relevant in the Neotropics, where bromeliad-associated frog diversity is high [19], and in Ecuador, where high-canopy tank bromeliads have been shown to support herpetofauna [9,20]. Because of the close relationship between bromeliads and amphibians, we consider bromeliad tanks as a possible source of Bd DNA detection and short-term environmental persistence [21,22].

Variation in forest strata and elevation may further influence amphibian habitat selection and Bd distribution patterns [23]. Bromeliads positioned in the canopy and subcanopy can differ in light exposure, which may influence leaf traits and tank conditions [24]. Because bromeliad morphology is closely associated with water holding capacity [25,26], vertical position within the forest may also affect the amount of water retained in bromeliad tanks. Elevational gradients are also associated with broad changes in temperature, precipitation and amphibian community composition [27]. In Ecuador, environmental susceptibility for Bd is expected to be greater at higher elevations and cooler temperatures [28]. However, recent work in lowland forests has shown that warm environments are not necessarily free of Bd [9], suggesting that local microhabitats may buffer or modify expected climatic patterns. Together, these observations indicate that Bd dynamics in tropical forests may depend not only on regional climate but also on how microhabitats are distributed vertically within forests and across elevations.

Bd dynamics may also vary among amphibian taxa because host ecology, habitat use and life history can influence both pathogen exposure and susceptibility [9,13,29]. In communities where bromeliads are common, these ecological differences may help explain why some amphibian taxa show higher Bd prevalence than others, even within the same landscape. Evaluating taxon-specific patterns of Bd infection alongside potential environmental pathogen reservoirs such as bromeliads can therefore offer a more comprehensive understanding of how environmental detection and host infection are connected across tropical forest communities.

In this study, we screened amphibian communities and bromeliad tanks for Bd across an elevational gradient in Ecuador, with an emphasis on vertical forest strata. Specifically, we sampled bromeliads at different heights in the canopy and surveyed co-occurring amphibian communities to compare patterns of Bd detection in bromeliad tanks with Bd infection in amphibian hosts. We assessed whether bromeliad-associated microhabitats may contribute to Bd persistence in these ecosystems and whether canopy height, bromeliad structural traits, elevation, host taxonomic identity and local climatic conditions help explain variation in Bd distribution patterns.

Tank bromeliads are used by amphibians for shelter, reproduction and larval development. Thus, we predicted that canopy bromeliads would have greater Bd prevalence than subcanopy bromeliads if amphibians increasingly use canopy refugia when conditions on the forest floor become less favourable, thereby increasing the likelihood of Bd deposition in canopy tank water. We also predicted that bromeliads from different forest strata would differ in structural traits related to water-holding capacity, because bromeliad morphology and tank conditions can vary with light exposure and vertical position within forests. Finally, given that elevation influences temperature, moisture and amphibian community composition, and that Bd infection varies among host taxa according to ecology, habitat use and susceptibility, we predicted that amphibian Bd prevalence would vary across elevation and host taxa. By integrating bromeliad-associated detection with host infection patterns, this study provides insight into how forest strata, elevation and host ecology shape Bd dynamics in tropical ecosystems.

## 2. Methodology

We used two complementary sampling approaches to assess Bd detection across forest strata and elevations in Ecuador: bromeliad tank content sampling and amphibian skin swabbing. Bromeliads were sampled at five sites and amphibians at eight sites, with five of those sites overlapping between datasets. As a result, the number of sites that we used varied depending on the analysis.

### 2.1. Bromeliad sampling

**Figure 1.**
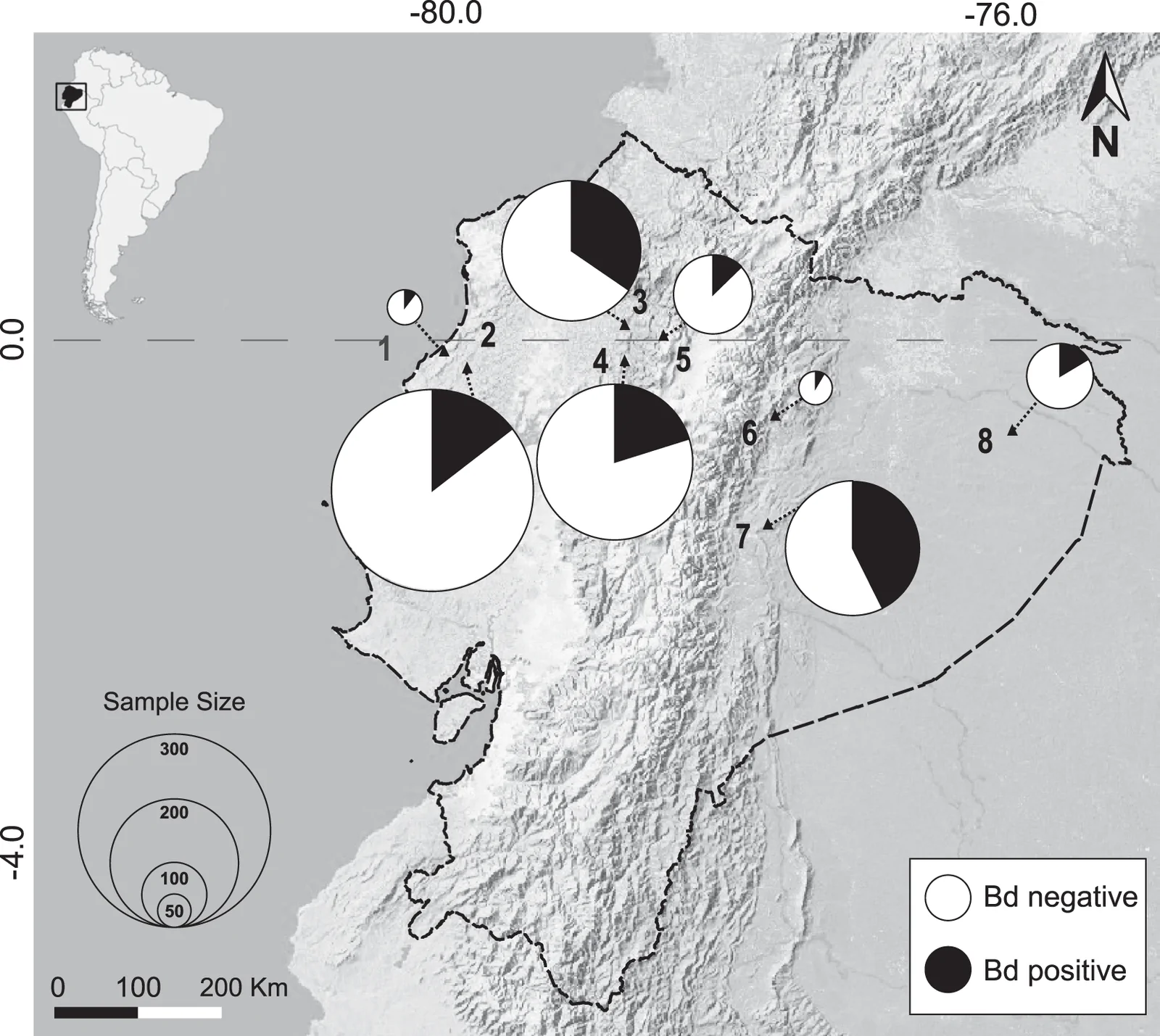
Map of sampling sites where 1263 amphibians were captured and tested for the fungus Bd in Ecuador. Pie charts at each location indicate the proportion of Bd-negative (white) and Bd-positive (black) amphibians. Sampling sites include: (1) Lalo Loor Dry Forest (*n* = 51), (2) Jama-Coaque Reserve (*n =* 302), (3) Mashpi Reserve (*n =* 209), (4) Saloya Private Land (*n =* 233), (5) Tandayapa Cloud Forest Station (*n =* 118), (6) Cosanga-Tamiaju Private Land (*n =* 50), (7) Zanja Arajuno Ecological Center (*n =* 202) and (8) Tiputini Biodiversity Station (*n =* 98). Pie size is proportional to the number of samples represented at each location.

We sampled at five sites spanning a broad elevational gradient in the Occidental montane and Amazonian forests of Ecuador from July 2022 to December 2023 (figure 1 and table 1). For clarity, we refer to these as low-elevation (Tiputini, 245 m), mid-elevation (Zanja Arajuno, 971 m and Saloya, 1103 m) and high-elevation (Cosanga, 2072 m and Tandayapa, 2248 m) sites.

**Table 1.** Geographic location, elevation, elevational category, sampling dates and sampling design for all sites included in this study of Bd across Ecuadorian forests. For each sampling site, the table shows latitude, longitude, elevation (m), elevational category (mid-elevation, low-elevation or high-elevation), sampling dates and whether bromeliads and amphibians were sampled. Repeated entries indicate sites visited during multiple sampling periods with different combinations of sample sources. Abbreviations: lat = latitude, long = longitude, m = metres, brom = bromeliads, amph = amphibians and mid = mid-elevation.

site	lat	long	elevation (m)	elevational category	sampling dates	brom sampled	amph sampled
Lalo Loor	–0.0772	–80.1533	36	low	June 2022	no	yes
Tiputini	–0.6741	–76.3972	245	low	June 2023	yes	yes
Jama-Coaque	–0.1161	–80.1234	508	low	June 2021; December 2021; June 2022; July 2022	no	yes
Mashpi	0.1659	–78.8782	839	mid	July 2021	no	yes
Zanja Arajuno	–1.3316	–77.8764	971	mid	December 2023	yes	yes
Saloya	0.0167	–78.9003	1103	mid	July 2022	no	yes
Saloya	0.0167	–78.9003	1103	mid	January 2023	yes	yes
Cosanga	–0.5789	–77.8668	2072	high	June 2023	yes	yes
Tandayapa	–0.015	–78.6859	2248	high	December 2022	yes	yes

To survey canopy bromeliads, we used single-rope climbing techniques following the methodology developed by McCracken and Forstner [30]. This approach uses a single-rope ascent system to access the forest canopy. We used a slingshot to set a leader line in the trees, which allowed the climber to ascend using mechanical knee and foot ascenders.

At each site, we used sterile syringes to collect 4 ml of homogenized bromeliad content, including water and suspended organic material, from 30 bromeliads in the canopy (greater than 4 m above ground) and 30 bromeliads in the subcanopy (4 m or less above ground), yielding a total of 300 bromeliads sampled across the five sampling sites. Prior to sampling each bromeliad, we approached the plant without physical contact and filled a single tube with 2 ml of nuclease-free water, serving as a negative control. All bromeliads were handled and sampled with powder-free nitrile examination gloves (Micro Flex®, Reno, NV, USA), which were replaced after each bromeliad.

In the field, sample material and negative controls were preserved by centrifuging the tubes for 10 min at 6000 r.p.m. (Heathrow Scientific®, Vernon Hills, IL, USA), removing the supernatant, and adding 1.2 ml of 95% ethanol to the remaining pellet. Although not all sampled bromeliads could be identified to species, all were tank bromeliads belonging to the genera *Tillandsia, Aechmea* and *Guzmania*. We characterized each bromeliad by measuring: (i) height above ground (measured in metres; MAG = metres above ground), (ii) bromeliad height (measured in cm from the base to the highest standing leaf), (iii) bromeliad width (measured in cm from outer leaf to opposite outer leaf) and (iv) base width (measured in cm). All measurements were recorded using a long measuring tape (model 34–760; Stanley Black & Decker, New Britain, CT, USA).

### 2.2. Amphibian sampling

We sampled amphibian communities at eight sites spanning Coastal, Occidental montane and Amazonian forests of Ecuador from June 2021 to December 2023, five of which were also sampled for bromeliads (figure 1 and table 1). For consistency with the bromeliad analyses, we refer to these as low-elevation (Lalo Loor Dry Forest, 36.1 m; Jama-Coaque Reserve (JCR), 508 m; Tiputini, 245 m), mid-elevation (Mashpi Reserve, 838.8 m; Zanja Arajuno, 971 m; Saloya, 1103 m) and high-elevation (Cosanga, 2072 m; Tandayapa, 2248 m) sites.

We surveyed amphibian communities using visual encounter surveys. At each site, four researchers systematically searched for amphibians during 5-hour nocturnal surveys over a 7-day sampling period. Upon capture, individuals were temporarily placed in clean, single-use plastic bags to minimize handling stress and prevent cross-contamination. During all handling procedures, researchers wore clean, powder-free nitrile examination gloves (Micro Flex®, Reno, NV, USA), changing gloves between each amphibian to maintain biosecurity.

All amphibians were morphologically identified and swabbed with a sterile dry rayon swab (Medical Wire, MW113, Corsham, Wiltshire, UK) following standardized Bd sampling procedures described by Hyatt *et al.* [31], consisting of 10 passes along the venter and five passes on the underside of each foot. Swabs were immediately placed in 1.5 ml sterile screw-top centrifuge tubes (VWR, 76417-208, Allentown, PA, USA) and later stored at –20°C until processing.

Amphibian sampling was conducted under authorization from the Ecuadorian Ministry of the Environment, Water and Ecological Transition (Permit No. MAE-DNB-CM-2018-0105 and MAE-DBI-DBI-CM-2022-0222) and in compliance with Institutional Animal Care and Use Committee (IACUC) protocols by Texas State University (Rodriguez-Protocol No. 8246).

### 2.3. eDNA detection of *Batrachochytrium dendrobatidis* in bromeliads

For bromeliad water samples, we first centrifuged the previously preserved samples for 5 min at 4000 r.p.m., then removed the ethanol supernatant, leaving all organic material at the bottom. To prevent DNA extraction inhibition caused by ethanol residue, we rinsed the pellets twice with 1 ml of DNase/RNase-Free Deionized Water (ThermoFisher, Waltham, MA, USA). We extracted DNA using the E.Z.N.A. Soil DNA Kit (Omega Bio-tek, Norcross, GA, USA), with the following modifications to the manufacturer's protocol: during the lysis step, we used a 3450 r.p.m. Mini-Beadbeater (BioSpec Products, Bartlesville, OK, USA) and 0.1 mm diameter glass beads (BioSpec Products, Bartlesville, OK, USA) to physically lyse all soil particles. During the final step of the extraction protocol, we eluted the DNA in 50 µl of elution buffer.

Preliminary testing showed that bromeliad water DNA extracted with the E.Z.N.A. Soil DNA Kit did not require dilution before quantitative polymerase chain reaction (qPCR), because these samples had low levels of inhibitors and target sequence, and diluting them caused the target concentration to fall below the qPCR detection range. We ran qPCRs on a QuantStudio 5 instrument (Applied Biosystems, Inc., Foster City, CA, USA) using the same master mix and standards as amphibian DNA. The cycling conditions were as follows: 2 min at 50°C, 10 min at 95°C, followed by 15 s at 95°C and 1 min at 60°C for 50 cycles [32]. Bromeliads were considered positive only if two of four qPCR replicates amplified above the lowest standard and the negative control for each bromeliad tested negative.

### 2.4. Detection of *Batrachochytrium dendrobatidis* on amphibian skin

We extracted DNA from all amphibian skin swabs using 50 µl of PrepMan Ultra (Applied Biosystems, Inc., Foster City, CA, USA), following the protocol described by Becker et al. [33], and diluted it 1 : 10 to limit reaction inhibition. To analyse extracted DNA, we used qPCR TaqMan Assays (Applied Biosystems, Inc., Foster City, CA, USA) with primers targeting the ITS-1 and 5.8S rRNA regions of Bd [32,34]. All qPCRs were performed in duplicate in 12.5 µl volumes using the Magnetic Induction Cycler (Bio Molecular Systems, Jersey City, NJ, USA).

Reactions included 6.25 µl 2× TaqMan Fast Advanced Master Mix (Applied Biosystems, Inc., Foster City, CA, USA), 1.38 µl nuclease-free water, 0.45 µM of each primer, 0.125 µM of probe, 200 ng bovine serum albumin (BSA) and 2.5 µl of diluted extracted DNA (variable concentrations). For the standards, we used a dynamic range from 0.5 to 5000 genomic equivalents of the isolate JEL423 (Bd-Global Panzootic Lineage) to quantify Bd infection loads. For negative controls, we substituted DNA with nuclease-free water. We used the following cycling conditions: initial denaturation at 95°C for 5 s, annealing starting at 60°C for 10 s and extension at 72°C for 10 s for 50 cycles. Samples were deemed positive if at least one replicate exhibited amplification above the lowest standard (0.5 genomic equivalents).

### 2.5. Climatic data collection

To investigate the relationships between climatic variables and Bd prevalence in amphibians and bromeliads, we used the Google Earth Engine (GEE) platform to extract temperature (°C) and precipitation (mm) for each sampling site during the corresponding sampling periods. Monthly mean temperature (°C) was calculated using the ERA5-Land hourly dataset from the ECMWF Climate Reanalysis. Precipitation was quantified as the total monthly precipitation for each sampling site using the UCSB-CHG CHIRPS Daily dataset. Precipitation was calculated by summing daily rainfall values within polygons representing each sampling site for each month. The GEE script used for these calculations is provided in the supplementary material.

We then summarized Bd observations by site, year, month and sample source (amphibian, canopy bromeliads, subcanopy bromeliads and all bromeliads). For amphibians, we calculated the total number of Bd-positive and Bd-negative individuals for each site-month-year combination using all available amphibian observations. For bromeliads, we calculated equivalent counts separately for canopy bromeliads, subcanopy bromeliads and total bromeliads pooled across strata. Amphibian and bromeliad count datasets were then merged with the climatic dataset by site, year and month using the left_join() function in the dplyr package in R.

### 2.6. Statistical analysis

Here, Bd status refers to whether a sample tested positive or negative for Bd (0 = negative and 1 = positive), Bd prevalence to refer to the proportion of Bd-positive samples (amphibians or bromeliads) among the total number of samples collected, with 95% binomial confidence intervals estimated using the binom package in R [35], and Bd intensity to refer to the qPCR-derived Bd load for positive samples. To reduce biases caused by under-sampled groups, only amphibian genera with at least 10 sampled individuals were included in subsequent analyses.

#### 2.6.1. Sources of *Batrachochytrium dendrobatidis*

To analyse the association between Bd sources (amphibians, canopy bromeliads and subcanopy bromeliads) and Bd status, we used a generalized linear mixed model with a binomial error distribution (‘lme4’ package in R). The response variable was Bd status (0 = negative and 1 = positive), and the fixed effect was Bd source. To account for potential non-independence among samples collected at the same site, we included sampling sites as a random effect. The model was fitted using the ‘glmer’ function in R. We then performed pairwise comparisons of the predicted probabilities of Bd-positive status among the three Bd sources using estimated marginal means with Tukey-adjusted *p*-values (‘emmeans’ package in R). To ensure comparability among Bd sources, we included only sampling sites that were sampled for all Bd sources within the same field season. As a result, we excluded data from Saloya (2022), JCR, Mashpi and Lalo Loor Dry Forest, where only amphibian data were collected.

#### 2.6.2. *Batrachochytrium dendrobatidis* in bromeliads

To assess whether bromeliads in the canopy differ in structural traits compared with those found in the subcanopy, we fit linear mixed-effect models with vertical distribution (canopy versus subcanopy) as a fixed effect and sampling site as a random effect. Specifically, we modelled bromeliad height, width and base diameter using the ‘lmer’ function (‘lme4’ and ‘lmerTest’ packages in R). The mixed-effects model for bromeliad width produced a singular fit because the variance attributed to sampling site was zero. We therefore analysed bromeliad width using a linear model without the random effect of site.

To assess the variation in Bd status in bromeliads across sampling sites, we fit a generalized linear model (GLM; ‘stats’ package in R) with a binomial error distribution and logit link using the ‘glm’ function. The response variable was Bd status, and sampling site was included as a fixed effect. We then tested whether Bd prevalence varied across the elevational gradient by fitting additional binomial GLMs using elevation (high, mid and low) as the predictor. For the elevation analyses, we summarized the number of Bd-positive and Bd-negative samples within each elevation and modelled these counts using a binomial error distribution. Separate elevation models were fit for total bromeliads, canopy bromeliads and subcanopy bromeliads.

#### 2.6.3. *Batrachochytrium dendrobatidis* on amphibians

To assess variation in Bd infection status in amphibians across taxonomic groups and sampling sites, we first filtered the dataset to include only amphibian genera represented by at least 10 sampled individuals (‘dplyr’ package in R). We then fit separate GLMs with a binomial error distribution and a logit link. One model included genus and sampling site as predictors, and a second included family and sampling site as predictors. To assess the overall significance of amphibian taxonomic identity and sampling site in the first two GLMs, we used likelihood-ratio tests with the drop1 function in R, comparing the full model against reduced models in which each predictor was removed one at a time. To compare the relative fit of amphibian infection-status models, we used Akaike's information criterion (AIC), with lower AIC values indicating better fit among models fitted to the same dataset and response variable. To assess spatial variation independently of amphibian taxonomy, we fit two additional GLMs: one with sampling site as the sole predictor and one with elevation as a categorical variable (high, mid and low).

To further investigate Bd infection across different taxonomic groups and sampling sites, we analysed variation in Bd infection intensity. Bd infection intensity was calculated as the arithmetic mean of the two qPCR technical replicates for each sample and used as the sample-level estimate of infection load in subsequent analyses. We fit two GLMs with Gaussian error distributions, using Bd load (log10-transformed) from Bd-positive individuals as the response variable; the first model included genus and sampling site as predictors, while the second included family and sampling site.

#### 2.6.4. Climatic data analysis

To test climatic effects on Bd prevalence, we fit binomial GLMs using the number of Bd-positive and Bd-negative samples as the response variable. Mean temperature and total monthly precipitation were standardized (mean = 0 and s.d. = 1) prior to analysis to improve model stability and allow direct comparison of effect sizes. For each source group, we fit one model including linear effects of temperature and precipitation and a second model including a quadratic temperature term in addition to precipitation. We compared candidate models using AIC, with lower AIC values indicating a better fit. Separate models were fit for amphibians, canopy bromeliads, subcanopy bromeliads and total bromeliads. All statistical analyses were conducted in R version 4.5.2 (31 October 2025) [36], and data visualization was performed using ‘ggplot2’ [37].

## 3. Results

### 3.1. *Batrachochytrium dendrobatidis* status among sample sources

**Figure 2.**
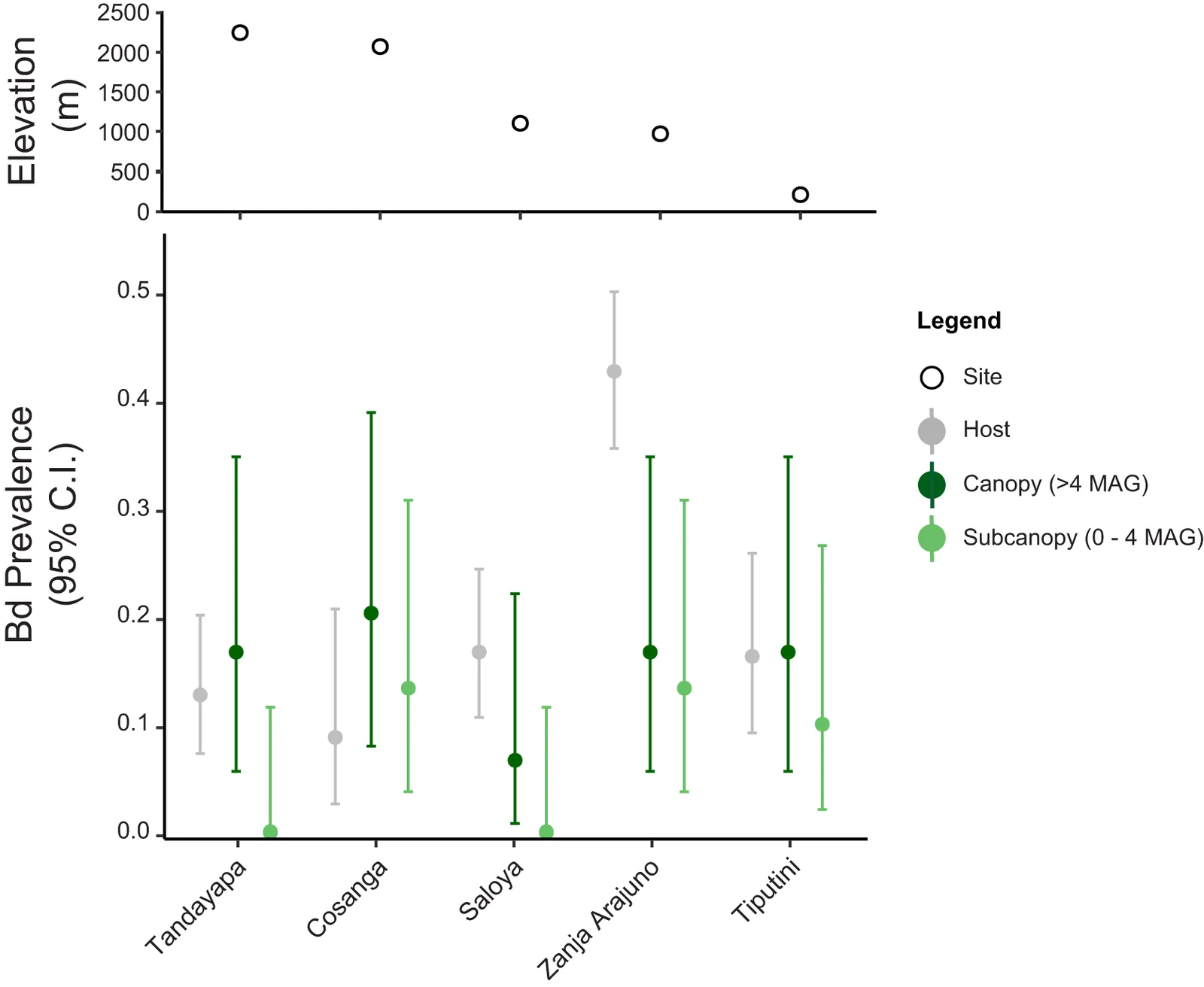
Mean prevalence of Bd infection in three different sample types (canopy bromeliads [dark green; 19.6 ± 8.5 MAG], subcanopy bromeliads [light green; 1.2 ± 1 MAG] and amphibian hosts [grey]) across five sampling sites in Ecuador: Tandayapa, Cosanga, Saloya, Zanja Arajuno and Tiputini. Error bars represent the 95% confidence intervals for each prevalence estimate. MAG = metres above ground.

Bd presence differed among Bd sources (figure 2). Relative to amphibians, subcanopy bromeliads showed a significantly lower probability of Bd-positive status (*β* = –1.18 ± 0.32, *z* = –3.69, *p* < 0.001), whereas canopy bromeliads did not differ significantly from amphibians (*β* = –0.48 ± 0.26, *z* = –1.86, *p* = 0.063). Pairwise comparisons confirmed that only subcanopy bromeliads had a significantly lower probability of Bd-positive status than amphibians (Tukey-adjusted *p* = 0.0007), whereas canopy bromeliads did not differ significantly from amphibians (*p* = 0.1501) or from subcanopy bromeliads (*p* = 0.1584).

### 3.2. Bromeliad morphology and *Batrachochytrium dendrobatidis*

Bromeliad height did not differ between canopy and subcanopy strata (canopy mean = 75.8 cm; *β* = –0.74 ± 4.69, *t* = –0.16, *p* = 0.874). Bromeliad width also did not differ significantly between canopy and subcanopy bromeliads (canopy mean = 104.4 cm; *β* = 7.66 ± 5.71, *t* = 1.34, *p* = 0.181). By contrast, subcanopy bromeliads had significantly narrower bases than canopy bromeliads (canopy mean = 10.1 cm; *β* = –1.74 ± 0.69, *t* = –2.54, *p* = 0.0117).

Bd presence in bromeliads varied among sampling sites. Saloya showed a lower probability of Bd-positive status (*β* = –1.76 ± 0.80, *z* = –2.20, *p* = 0.028), whereas Tandayapa (*β* = –0.79 ± 0.58, *z* = –1.36, *p* = 0.175), Tiputini (*β* = –0.26 ± 0.51, *z* = –0.51, *p* = 0.610) and Zanja Arajuno (*β* = –0.13 ± 0.50, *z* = –0.25, *p* = 0.803) did not show significant differences. Bromeliad Bd prevalence did not differ significantly across elevation categories. In the total bromeliad model, neither low-elevation sites (*β* = 0.07 ± 0.47, *z* = 0.16, *p* = 0.875) nor mid-elevation sites (*β* = –0.35 ± 0.42, *z* = –0.83, *p* = 0.408) differed significantly from high-elevation sites. No significant elevational differences were detected when canopy and subcanopy bromeliads were analysed separately. Bromeliad Bd patterns and morphology analyses were based on 300 bromeliads sampled across five sites.

### 3.3. *Batrachochytrium dendrobatidis* patterns across amphibian taxonomy, site and elevation

**Figure 3.**
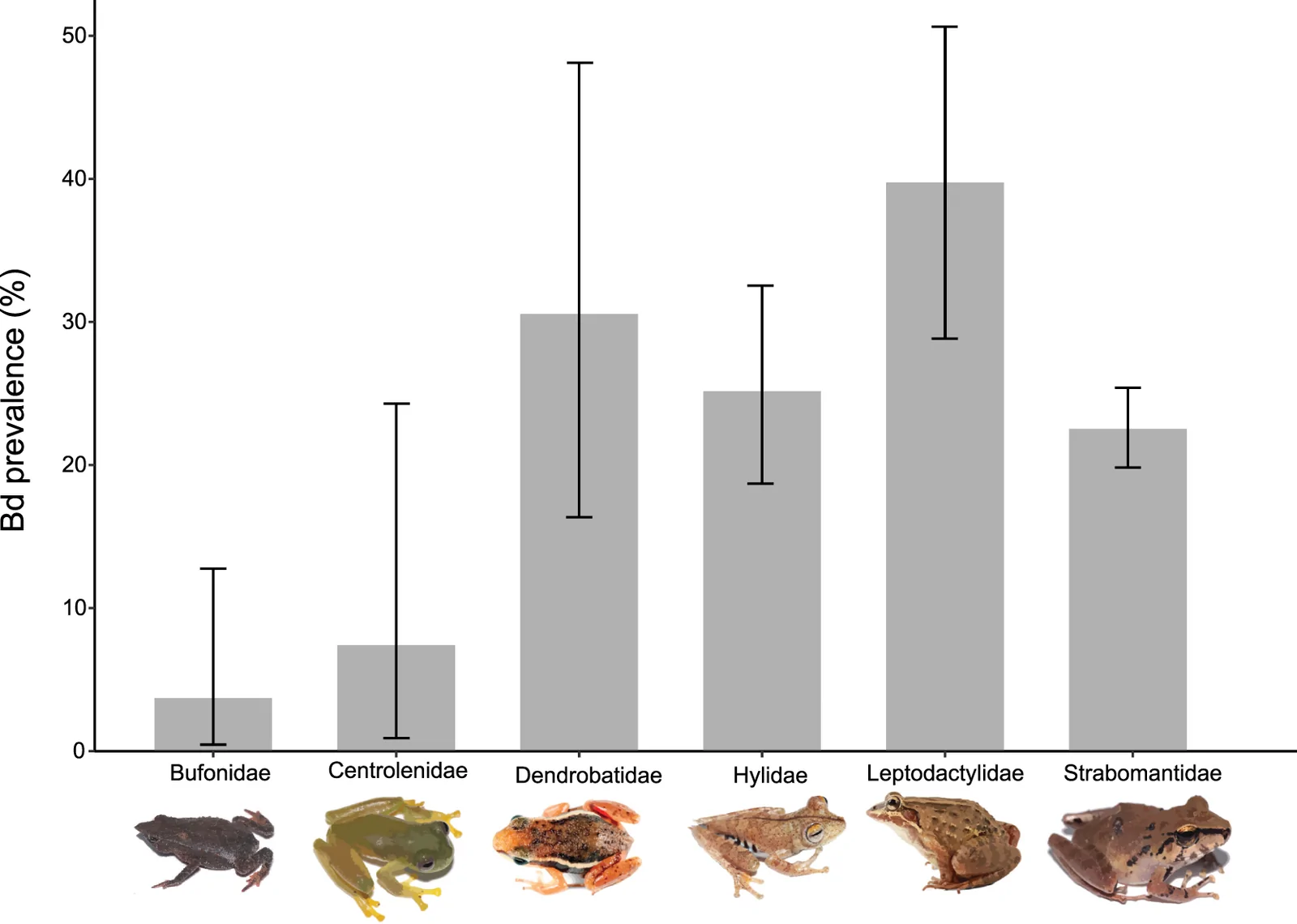
Prevalence of Bd infection across amphibian host families. Error bars indicate 95% confidence intervals of prevalence estimates. Only families represented in the dataset after restricting amphibian genera to those with at least 10 sampled individuals are shown. Representative species are depicted (not to scale). From left to right: *Rhinella festae*, *Rulyrana* sp., *Epipedobates machalilla*, *Boana appendiculata, Leptodactylus* sp. and *Pristimantis* sp.

Overall Bd prevalence in amphibians was 23% (95% CI = 20.7–25.5%) in amphibian hosts (*n* = 1263 from eight sampling sites; figures 2 and 3). Bd infection status in amphibians varied across taxonomic groups, sampling sites and elevation. In the family + sampling site model, family was a significant predictor of Bd-positive status. Relative to Bufonidae, Dendrobatidae, Leptodactylidae and Strabomantidae showed higher probability of Bd-positive status in Tukey-adjusted pairwise comparisons, whereas Hylidae and Centrolenidae did not differ significantly from Bufonidae after multiple-comparison correction (supplementary material, table S1). Among the first two models, the genus + sampling site model showed a better fit (AIC = 1207.4) than the family + sampling site model (AIC = 1216.9). In the genus + site model, *Phyllomedusa* and *Rhinella* showed lower Bd-positive probability than the reference genus, *Ameerega*. However, Tukey-adjusted pairwise comparisons among genera were more conservative, and only a small number of contrasts remained significant, primarily involving *Rhinella*, which had a lower Bd-positive probability than *Dendropsophus*, *Engystomops* and *Scinax* (supplementary material, table S2)*.*

Spatially, amphibians from Mashpi (*β* = 1.73 ± 0.54, *z* = 3.19, *p* = 0.001) and Zanja Arajuno (*β* = 2.08 ± 0.54, *z* = 3.83, *p* < 0.001) showed significantly higher probability of Bd-positive status than Cosanga, while Saloya showed a marginal positive trend (*β* = 1.00 ± 0.55, *z* = 1.83, *p* = 0.068). These sites all fall within the mid-elevation category, and the elevation model similarly showed that mid-elevation sites had significantly higher probability of Bd-positive status than the high-elevation sites (*β* = 1.27 ± 0.26, *z* = 4.90, *p* < 0.001), whereas low-elevation sites did not differ significantly (*β* = 0.26 ± 0.28, *z* = 0.92, *p* = 0.357; figure 2). Amphibian Bd patterns across taxonomy, sampling site and elevation were modelled, including 1211 observations across eight sites**,** after restricting the dataset to genera represented by at least 10 sampled individuals. Regarding Bd infection intensity, we found no significant differences in Bd infection loads across sampling sites or amphibian taxonomic identities, in either the genus- or family-level models.

### 3.4. Climatic analysis of *Batrachochytrium dendrobatidis* prevalence

For all bromeliads (300 bromeliad samples), neither temperature nor precipitation significantly predicted Bd prevalence in the linear model. Although the quadratic temperature model had a lower AIC than the linear model (28.44 versus 31.23), no climatic predictor was significant. In the quadratic model, precipitation remained non-significant (*β* = –0.51 ± 0.73, *z* = –0.70, *p* = 0.483), the linear temperature term was non-significant (*β* = 0.43 ± 0.67, *z* = 0.64, *p* = 0.522) and the quadratic temperature term showed only a marginal trend (*β* = 1.00 ± 0.51, *z* = 1.95, *p* = 0.051).

When we analysed the bromeliad data by stratum, climatic variables did not significantly predict Bd prevalence in either canopy (five site-month observations, representing 150 bromeliad samples) or subcanopy strata (five site-month observations, representing 150 bromeliad samples). In canopy bromeliads, the linear model showed no significant effects of temperature (*β* = 0.10 ± 0.68, *z* = 0.14, *p* = 0.889) or precipitation (*β* = –0.13 ± 0.68, *z* = –0.19, *p* = 0.847). Although the quadratic temperature model had a slightly lower AIC than the linear model (24.03 versus 26.42), none of the predictors were significant (precipitation: *β* = –1.25 ± 0.95, *z* = –1.32, *p* = 0.186; linear temperature term: *β* = 1.00 ± 0.87, *z* = 1.15, *p* = 0.250; quadratic temperature term: *β* = 1.30 ± 0.72, *z* = 1.80, *p* = 0.073). Similarly, in subcanopy bromeliads, the linear model showed no significant effects of temperature (*β* = –1.25 ± 1.10, *z* = –1.14, *p* = 0.255) or precipitation (*β* = 1.66 ± 1.21, *z* = 1.36, *p* = 0.173), and the quadratic model was not better supported by AIC (23.94 versus 22.55). None of the predictors in the quadratic subcanopy model were significant (precipitation: *β* = 1.04 ± 1.40, *z* = 0.75, *p* = 0.457; linear temperature term: *β* = –0.78 ± 1.22, *z* = –0.64, *p* = 0.521; quadratic temperature term: *β* = 0.53 ± 0.72, *z* = 0.74, *p* = 0.460).

In amphibians (12 site-month observations, representing 1211 amphibian samples), climatic conditions during the sampling month were significantly associated with Bd prevalence. The linear model showed significant positive relationships between Bd prevalence and both precipitation (*β* = 0.14 ± 0.06, *z* = 2.24, *p* = 0.025) and temperature (*β* = 0.17 ± 0.08, *z* = 2.00, *p* = 0.046). However, the quadratic temperature model showed much stronger support based on AIC (92.89) than the linear model (136.07). In the better-supported model, Bd prevalence increased with precipitation (*β* = 0.23 ± 0.07, *z* = 3.47, *p* < 0.001) and showed a significant nonlinear association with temperature (linear term: *β* = –0.31 ± 0.12, *z* = –2.65, *p* = 0.008; quadratic term: *β* = –0.91 ± 0.15, *z* = –5.97, *p* < 0.001).

## 4. Discussion

### 4.1. Environmental reservoirs of *Batrachochytrium dendrobatidis*

We detected Bd in canopy and subcanopy bromeliads across tropical forests in Ecuador, supporting the concept that bromeliad tanks can function as viable microhabitats for Bd detection and allow short-term environmental pathogen persistence in forest canopies. Although Bd prevalence was higher in canopy than in subcanopy bromeliads, this difference was not statistically significant. Canopy bromeliads did, however, have significantly wider bases than subcanopy bromeliads, a trait that may be associated with greater water-holding capacity [38] and longer persistence of water sources during times of low rainfall. In canopy environments, where humidity fluctuates more widely than in subcanopy habitats [39,40], bromeliads may provide limited aquatic refuge for amphibians [41]. Previous studies have shown that amphibians do not occupy microhabitats randomly but instead select them based on traits such as size, vertical positioning and water-holding capacity [42–44]. Although we did not inspect all sampled bromeliads for amphibians to minimize disturbance, canopy-dwelling amphibians using these plants for refuge or reproduction are probably exposed to Bd through these water repositories. Together, these results suggest that microhabitat structure and vertical positioning may influence Bd dynamics. However, at a broader spatial scale bromeliad Bd prevalence did not differ significantly across elevation categories, suggesting that bromeliad-associated Bd detection may be less structured by the elevational gradient than amphibian host infection patterns.

### 4.2. Environmental dynamics of *Batrachochytrium dendrobatidis*

Among amphibians, the probability of Bd-positive status was significantly higher in Mashpi (838.8 m) and Zanja Arajuno (971 m), with Saloya (1103 m) showing a marginal positive trend. Together, these results indicate that the probability of Bd-positive status in amphibians is higher at mid-elevation sites rather than increasing steadily with elevation. Although previous work identified Andean high-elevation regions of Venezuela, Colombia and Ecuador as highly susceptible to Bd establishment [45], our findings suggest that, within Ecuador, Bd risk peaks at intermediate elevational ranges.

This mid-elevation pattern may reflect the combined influence of local climate and amphibian community composition. Climatic analyses showed that amphibian Bd prevalence increased with precipitation and followed a nonlinear relationship with temperature, suggesting that the higher Bd prevalence observed at mid-elevation sites may reflect optimal intermediate thermal conditions rather than a simple pattern of increasing Bd prevalence with elevation or cooler temperatures.

### 4.3. Host identity and *Batrachochytrium dendrobatidis*

Overall, the probability of being Bd-positive was higher in the families Strabomantidae, Dendrobatidae and Leptodactylidae than in Bufonidae, while Hylidae showed the same general trend but was not significant after Tukey adjustment (supplementary material, table S1). At the genus scale, model coefficients suggested lower Bd-positive probability in genera such as *Rhinella* and *Phyllomedusa*, pointing to possible genus-specific differences in exposure and susceptibility. *Rhinella* species are predominantly terrestrial, which may reduce contact with aquatic microhabitats where Bd is detected, whereas *Phyllomedusa* may also differ in habitat use or skin defences in ways that reduce infection probability (supplementary material, table S2). These patterns suggest that host ecology may help explain finer-scale variation in Bd dynamics across amphibian communities.

Interestingly, all 897 specimens collected from the family Strabomantidae were direct-developing species belonging to the genus *Pristimantis*. This aligns with previous research reporting higher Bd prevalence in *Pristimantis* on the western slopes of the Andes compared with species from the families Centrolenidae and Hylidae [13], possibly owing to differences in immune response efficacy [46]. Meanwhile, the high prevalence observed in Dendrobatidae (30.6%; 95% CI = 16.3–48.1), Hylidae (25.2%; 95% CI = 18.7–32.5) and Leptodactylidae (39.7%; 95% CI = 28.8–51.5) is consistent with the hypothesis that Bd transmission is amplified in aquatic environments, given that members of these families have a tadpole stage and rely heavily on water bodies [47,48]. This pattern aligns with broader amphibian population declines in the Andes [49], which have disproportionately affected species with aquatic larval development [50–52].

Conversely, we found significantly lower Bd prevalence in the genus *Rhinella* (3.7%; 95% CI = 0.5–12.7), consistent with previous studies that have identified this genus as less susceptible to Bd infection [53]. Species of this genus are predominantly terrestrial, which probably reduces their exposure to aquatic microhabitats where Bd is detected. This genus-specific resistance supports the hypothesis that amphibians exhibit distinct physiological or behavioural defences against Bd [54,55]. These findings highlight the complex dynamics of Bd in highly diverse ecosystems and the site- and host-specific immune responses that different populations have developed to mitigate infection. Simultaneously, as hosts and pathogens co-evolve, Bd has also developed strategies to enhance its dispersal and virulence [56].

### 4.4. Patterns of pathogen endemism

We observed no clinical signs of chytridiomycosis in any amphibian sampled. Outbreaks of chytridiomycosis are spatio-temporally cyclic and clustered [57–59], which might explain the absence of symptomatic hosts at our sites. Additionally, hosts in this region probably experienced historic declines driven by chytridiomycosis [60], which may have resulted in immunological tolerance to infection by this pathogen post-invasion [59,61,62]. While initial high infection loads are typically associated with chytridiomycosis outbreaks [61], we detected low infection loads among Bd-positive amphibians across samples and sites. Our data and other contemporary detections of Bd provide evidence that Bd is widespread and enzootic across Ecuador. This pattern is consistent with retrospective studies leveraging museum specimens showing Bd was present in South America since the late 1800s to early 1900s [33,49,63]. Experimental studies assessing the current virulence of Bd in this region and population-scale retrospective surveys in Ecuador would be valuable in understanding these patterns.

## 5. Conclusion

Taken together, these results suggest that bromeliads and amphibians do not respond to the same drivers at the same scale. Bd detection in bromeliads showed weak differences across strata, sites and elevation, whereas amphibian Bd detection status varied more clearly across host taxa, elevation and climatic conditions. This contrast suggests that environmental detection of Bd and host infection patterns are related, but not interchangeable components of pathogen dynamics in tropical forests. Our study suggests that amphibian Bd risk may peak at intermediate elevational ranges, an elevational band that overlaps with broader montane regions where amphibian declines and Bd-associated losses have been repeatedly documented in the Neotropics and Andes. These results underscore the complexity of Bd transmission in tropical ecosystems and suggest that microhabitat characteristics, amphibian diversity and local environmental conditions should be considered when assessing Bd risks across highly diverse ecosystems.

## Supplementary Material



## Data Availability

The supplementary material contains all data supporting this study's findings [64]. The datasets include the identification of collected amphibians, Bd screening results and ecological data for each collected bromeliad. The R code used for statistical analyses is also available in the supplementary material.

## References

[1] Scheele BC *et al.* 2019 Amphibian fungal panzootic causes catastrophic and ongoing loss of biodiversity. Science 363, 1459–1463. (doi:10.1126/science.aav0379) 30923224

[2] Azat C , Alvarado-Rybak M, Solano-Iguaran JJ, Velasco A, Valenzuela-Sánchez A, Flechas SV, Peñafiel‐Ricaurte A, Cunningham AA, Bacigalupe LD. 2022 Synthesis of *B**atrachochytrium dendrobatidis* infection in South America: amphibian species under risk and areas to focus research and disease mitigation. Ecography 2022, e05977. (doi:10.1111/ecog.05977)

[3] Bradley GA , Rosen PC, Sredl MJ, Jones TR, Longcore JE. 2002 Chytridiomycosis in native Arizona frogs. J. Wildl. Dis. 38, 206–212. (doi:10.7589/0090-3558-38.1.206) 11838218

[4] Forrest MJ , Schlaepfer MA. 2011 Nothing a hot bath won't cure: infection rates of amphibian chytrid fungus correlate negatively with water temperature under natural field settings. PLoS ONE 6, e28444. (doi:10.1371/journal.pone.0028444) 22205950 PMC3244395

[5] McDonald KR , Méndez D, Müller R, Freeman AB, Speare R. 2005 Decline in the prevalence of chytridiomycosis in frog populations in North Queensland, Australia. Pac. Conserv. Biol. 11, 114–120. (doi:10.1071/PC050114)

[6] Turner A , Wassens S, Heard G. 2021 Chytrid infection dynamics in frog populations from climatically disparate regions. Biol. Conserv. 264, 109391. (doi:10.1016/j.biocon.2021.109391)

[7] Marshall TL , Baca CR, Correa DT, Forstner MRJ, Hahn D, Rodriguez D. 2019 Genetic characterization of chytrids isolated from larval amphibians collected in central and east Texas. Fungal Ecol. 39, 55–62. (doi:10.1016/j.funeco.2018.12.001)

[8] Neely WJ , Moretta-Urdiales MDM, Smart U, Lynch RL, Guayasamin JM, McCracken SF, Rodriguez D. 2025 Community-wide genotyping of *B**atrachochytrium dendrobatidis* in Ecuadorian forests. EcoHealth. (doi:10.1007/s10393-025-01716-y) PMC13582374 40325318

[9] Moretta-Urdiales M , Narváez AE, Barreno M, Cuadrado S, Molina-Moreira N, Neely WJ, Guayasamin JM, Rodriguez D. 2026 Contrasting seasonal prevalence of *Batrachochytrium dendrobatidis* on anurans in fragmented urban forests. J. Wildl. Dis. 62, 63–75. (doi:10.7589/jwd-d-25-00033) 41412158

[10] Smart U , McCracken SF, Brunner RM, Rivera C, Rodriguez D. 2024 Detection of the *Batrachochytrium dendrobatidis* global panzootic lineage in Ecuadorian anurans of the Amazonian lowlands. Dis. Aquat. Organ. 160, 115–125. (doi:10.3354/dao03830) 39665309

[11] Narváez-Narváez DA , Cabrera-Andrade A, Merino-Viteri A, Paz-y-Miño C, Burgos G, Genoy-Puerto A. 2021 Infection dynamics of *B**atrachochytrium dendrobatidis* in two frog species inhabiting Quito's Metropolitan Guangüiltagua Park, Ecuador. J. Wildl. Dis. 57, 749–760. (doi:10.7589/jwd-d-20-00110) 34525187

[12] McCracken S , Gaertner JP, Forstner MRJ, Hahn D. 2009 Detection of *B**atrachochytrium dendrobatidis* in amphibians from the forest floor to the upper canopy of an Ecuadorian Amazon lowland rainforest. Herpetol. Rev. 40, 190–195.

[13] Guayasamin JM , Mendoza-Henao ÁM, Longo AV, Zamudio KR, Bonaccorso E. 2014 High prevalence of *Batrachochytrium dendrobatidis* in an Andean frog community (Reserva Las Gralarias, Ecuador). Amphib. Reptile Conserv. 8, 33–44.

[14] Donnelly MA . 1989 Effects of reproductive resource supplementation on space-use patterns in *Dendrobates pumilio*. Oecologia 81, 212–218. (doi:10.1007/BF00379808) 28312540

[15] Valencia-Díaz S , Flores-Palacios A, Rodríguez-López V, Ventura-Zapata E, Jiménez-Aparicio AR. 2010 Effect of host-bark extracts on seed germination in *Tillandsia recurvata*, an epiphytic bromeliad. J. Trop. Ecol. 26, 571–581. (doi:10.1017/S0266467410000374)

[16] Ruano-Fajardo G , Rovito SM, Ladle RJ. 2014 Bromeliad selection by two salamander species in a harsh environment. PLoS ONE 9, e98474. (doi:10.1371/journal.pone.0098474) 24892414 PMC4043640

[17] Peixoto OL . 2013 Associação de anuros a bromeliáceas na Mata Atlântica. Rev. Ciênc. Vida 17, 75–83.

[18] Wake DB . 1987 Adaptive radiation of salamanders in Middle American cloud forests. Ann. Mo. Bot. Gard. 74, 242–264. (doi:10.2307/2399397)

[19] Zocca C , Ghilardi-Lopes NP, Ferreira RB. 2024 Bromeliad-dwelling frogs revealed by citizen scientists. Diversity 16, 363. (doi:10.3390/d16070363)

[20] McCracken SF , Forstner MRJ. 2014 Oil road effects on the anuran community of a high canopy tank bromeliad (*Aechmea zebrina*) in the upper Amazon basin, Ecuador. PLoS ONE 9, e85470. (doi:10.1371/journal.pone.0085470) 24416414 PMC3885719

[21] Torresdal JD , Farrell AD, Goldberg CS. 2017 Environmental DNA detection of the golden tree frog (*Phytotriades auratus*) in bromeliads. PLoS ONE 12, e0168787. (doi:10.1371/journal.pone.0168787) 28052079 PMC5215732

[22] Lopes CM , Santos MTT, Baêta D, Sabbag AF, Haddad CFB. 2021 Environmental DNA as a non-invasive alternative for surveying aquatic communities in tank bromeliads. Mar. Freshw. Res. 74, 441–448. (doi:10.1071/mf20333)

[23] Stuckert AMM , Stone JP, Asper JR, Rinker MG, Rutt CL, Trimmer NC, Lindquist ED. 2009 Microhabitat use and spatial distribution in Picado's bromeliad treefrog, *Isthmohyla picadoi* (Anura, Hylidae). Phyllomedusa: J. Herpetol. 8, 125–134. (doi:10.11606/issn.2316-9079.v8i2p125-134)

[24] Lafont Rapnouil T , Gallant Canguilhem M, Julien F, Céréghino R, Leroy C. 2023 Light intensity mediates phenotypic plasticity and leaf trait regionalization in a tank bromeliad. Ann. Botany 132, 443–454. (doi:10.1093/aob/mcad126) 37647886 PMC10667009

[25] Zotz G , Thomas V. 1999 How much water is in the tank? Model calculations for two epiphytic bromeliads. Ann. Botany 83, 183–192. (doi:10.1006/anbo.1998.0809)

[26] Zotz G , Leja M, Aguilar-Cruz Y, Einzmann HJR. 2020 How much water is in the tank? An allometric analysis with 205 bromeliad species. Flora 264, 151557. (doi:10.1016/j.flora.2020.151557)

[27] Amador L , Soto-Gamboa M, Guayasamin JM. 2019 Integrating alpha, beta, and phylogenetic diversity to understand anuran fauna along environmental gradients of tropical forests in western Ecuador. Ecol. Evol. 9, 11 040–11 052. (doi:10.1002/ece3.5593) PMC6802013 31641453

[28] Menéndez-Guerrero PA , Graham CH. 2013 Evaluating multiple causes of amphibian declines of Ecuador using geographical quantitative analyses. Ecography 36, 756–769. (doi:10.1111/j.1600-0587.2012.07877.x)

[29] Lips KR , Reeve JD, Witters LR. 2003 Ecological traits predicting amphibian population declines in Central America. Conserv. Biol. 17, 1078–1088. (doi:10.1046/j.1523-1739.2003.01623.x)

[30] McCracken SF , Forstner MRJ. 2008 Bromeliad patch sampling technique for canopy herpetofauna in Neotropical forests. Herpetol. Rev. 39, 170–174.

[31] Hyatt AD *et al.* 2007 Diagnostic assays and sampling protocols for the detection of *B**atrachochytrium dendrobatidis*. Dis. Aquat. Organ. 73, 175–192. (doi:10.3354/dao073175) 17330737

[32] Boyle DG , Boyle DB, Olsen V, Morgan JAT, Hyatt AD. 2004 Rapid quantitative detection of chytridiomycosis (*Batrachochytrium dendrobatidis*) in amphibian samples using real-time Taqman PCR assay. Dis. Aquat. Organ. 60, 141–148. (doi:10.3354/dao060141) 15460858

[33] Becker CG , Rodriguez D, Lambertini C, Toledo LF, Haddad CFB. 2016 Historical dynamics of *Batrachochytrium dendrobatidis* in Amazonia. Ecography 39, 954–960. (doi:10.1111/ecog.02055)

[34] Garland S , Baker A, Phillott AD, Skerratt LF. 2010 BSA reduces inhibition in a TaqMan assay for the detection of *B**atrachochytrium dendrobatidis*. Dis. Aquat. Organ. 92, 113–116. (doi:10.3354/dao02053) 21268973

[35] Dorai-Raj S . 2014 binom: binomial confidence intervals for several parameterizations. R package version. https://cran.rstudio.com/web/packages/binom/binom.pdf.

[36] R Development Core Team. 2018 R: A language and environment for statistical computing. Vienna, Austria: R Foundation for Statistical Computing. https://www.R-project.org/.

[37] Wickham H . 2016 Programming with ggplot2. In Ggplot2: elegant graphics for data analysis, pp. 241–253. Cham, Switzerland: Springer.

[38] Richardson BA . 1999 The bromeliad microcosm and the assessment of faunal diversity in a Neotropical forest. Biotropica 31, 321–336. (doi:10.1111/j.1744-7429.1999.tb00144.x)

[39] Keppel G , Anderson S, Williams C, Kleindorfer S, O'Connell C. 2017 Microhabitats and canopy cover moderate high summer temperatures in a fragmented Mediterranean landscape. PLoS ONE 12, e0183106. (doi:10.1371/journal.pone.0183106) 28806772 PMC5555690

[40] Abera T , Heiskanen J, Maeda E, Odongo V, Pellikka P. 2023 Impacts of land cover and management change on top-of-canopy and below-canopy temperatures in Southeastern Kenya. Sci. Total Environ. 874, 162560. (doi:10.1016/j.scitotenv.2023.162560) 36870488

[41] dos Santos Protázio A , dos Santos Protázio A, de Siqueira Ribeiro E, de Souza Nogueira EM, de Moura GJB. 2013 The role of bromeliad architecture and abiotic factors in occupation by anurans. Neotrop. Biol. Conserv. 8, 88–95. (doi:10.4013/nbc.2013.82.04)

[42] Kam Y-C , Chuang Z-S, Yen C-F. 1996 Reproduction, oviposition-site selection, and tadpole oophagy of an arboreal nester, *Chirixalus eiffingeri* (Rhacophoridae), from Taiwan. J. Herpetol. 30, 52–59. (doi:10.2307/1564706)

[43] Mageski MM , Ferreira RB, Beard KH, Costa LC, Jesus PR, Medeiros CC, Ferreira PD. 2016 Bromeliad selection by *Phyllodytes luteolus* (Anura, Hylidae): the influence of plant structure and water quality factors. J. Herpetol. 50, 108–112. (doi:10.1670/14-166)

[44] de Oliveira FB , Navas CA. 2004 Plant selection and seasonal patterns of vocal activity in two populations of the bromeligen treefrog *S**cinax perpusillus* (Anura, Hylidae). J. Herpetol. 38, 331–339. (doi:10.1670/205-03A)

[45] Ron SR . 2005 Predicting the distribution of the amphibian pathogen *B**atrachochytrium dendrobatidis* in the New World. Biotropica 37, 209–221. (doi:10.1111/j.1744-7429.2005.00028.x)

[46] Rosenblum EB , Poorten TJ, Settles M, Murdoch GK, Robert J, Maddox N, Eisen MB. 2009 Genome-wide transcriptional response of *Silurana* (*Xenopus*) *tropicalis* to infection with the deadly chytrid fungus. PLoS ONE 4, e6494. (doi:10.1371/journal.pone.0006494) 19701481 PMC2727658

[47] Brem FMR , Lips KR. 2008 *Batrachochytrium dendrobatidis* infection patterns among Panamanian amphibian species, habitats and elevations during epizootic and enzootic stages. Dis. Aquat. Organ. 81, 189–202. (doi:10.3354/dao01960) 18998584

[48] Lips KR , Burrowes PA, Mendelson III JR, Parra-Olea G. 2005 Amphibian declines in Latin America: widespread population declines, extinctions, and impacts. Biotropica 37, 163–165. (doi:10.1111/j.1744-7429.2005.00023.x)

[49] Jervis PA , Sullivan C, Manzano-Pasquel A, Rosa GM, Tarvin RD, Ron SR, Vredenburg VT, Fisher MC, Merino‐Viteri A. 2026 Rapid emergence of an amphibian pathogen coincided with historic amphibian declines in the Neotropics. Divers. Distrib. 32, e70187. (doi:10.1111/ddi.70187)

[50] Coloma LA , Duellman WE, Almendáriz A, Ron S, Terán-Valdez A, Guayasamin JM. 2010 Five new (extinct?) species of Atelopus (Anura: Bufonidae) from andean Colombia, Ecuador, and Peru. Zootaxa 2574, 1–54. (doi:10.5281/zenodo.197448)

[51] Jervis P , Karlsdóttir B, Jehle R, Almeida-Reinoso D, Almeida-Reinoso F, Ron S, Fisher MC, Merino-Viteri A. 2020 Disease reservoirs threaten the recently rediscovered Podocarpus stubfoot toad (*Atelopus podocarpus*). Amphib. Reptile Conserv. 14, 157–164. (doi:10.5281/zenodo.16827671)

[52] La Marca E . 2005 Catastrophic population declines and extinctions in Neotropical harlequin frogs (Bufonidae: *Atelopus*). Biotropica 37,190–201. (doi:10.1111/j.1744-7429.2005.00026.x)

[53] Brannelly LA , Martin G, Llewelyn J, Skerratt LF, Berger L. 2018 Age- and size-dependent resistance to chytridiomycosis in the invasive cane toad *Rhinella marina*. Dis. Aquat. Organ. 131, 107–120. (doi:10.3354/dao03278) 30460917

[54] Van Rooij P , Martel A, Haesebrouck F, Pasmans F. 2015 Amphibian chytridiomycosis: a review with focus on fungus-host interactions. Vet. Res. 46, 137. (doi:10.1186/s13567-015-0266-0) 26607488 PMC4660679

[55] Bacigalupe LD , Soto-Azat C, García-Vera C, Barría-Oyarzo I, Rezende EL. 2017 Effects of amphibian phylogeny, climate and human impact on the occurrence of the amphibian-killing chytrid fungus. Glob. Change Biol. 23, 3543–3553. (doi:10.1111/gcb.13610) 28055125

[56] Carvalho T , Belasen AM, Toledo LF, James TY. 2024 Coevolution of a generalist pathogen with many hosts: the case of the amphibian chytrid *Batrachochytrium dendrobatidis*. Curr. Opin. Microbiol. 78, 102435. (doi:10.1016/j.mib.2024.102435) 38387210

[57] Carvalho T , Becker CG, Toledo LF. 2017 Historical amphibian declines and extinctions in Brazil linked to chytridiomycosis. Proc. R. Soc. B 284, 20162254. (doi:10.1098/rspb.2016.2254) PMC5310605 28179514

[58] Belasen AM *et al.* 2024 Chytrid infections exhibit historical spread and contemporary seasonality in a declining stream-breeding frog. R. Soc. Open Sci. 11, 231270. (doi:10.1098/rsos.231270) 38298390 PMC10827429

[59] Scheele BC *et al.* 2017 After the epidemic: ongoing declines, stabilizations and recoveries in amphibians afflicted by chytridiomycosis. Biol. Conserv. 206, 37–46. (doi:10.1016/j.biocon.2016.12.010)

[60] Ron SR , Merino A. 2000 Amphibian declines in Ecuador: overview and first report of chytridiomycosis from South America. Froglog 42, 2–3.

[61] Grogan LF , Mangan MJ, McCallum HI. 2023 Amphibian infection tolerance to chytridiomycosis. Phil. Trans. R. Soc. B 378, 20220133. (doi:10.1098/rstb.2022.0133) 37305912 PMC10258672

[62] Trujillo AL , Hoffman EA, Becker CG, Savage AE. 2021 Spatiotemporal adaptive evolution of an MHC immune gene in a frog-fungus disease system. Heredity 126, 640–655. (doi:10.1038/s41437-020-00402-9) 33510466 PMC8115231

[63] Rodriguez D , Becker CG, Pupin NC, Haddad CFB, Zamudio KR. 2014 Long-term endemism of two highly divergent lineages of the amphibian-killing fungus in the Atlantic Forest of Brazil. Mol. Ecol. 23, 774–787. (doi:10.1111/mec.12615) 24471406

[64] Moretta-Urdiales MDM , McCracken S, Guayasamin JM, Lynch RL, Tenorio M, Neely WJ, Rodriguez D. 2026 Supplementary material from: Evaluating bromeliads as environmental reservoirs for *Batrachochytrium dendrobatidis* and amphibian disease dynamics in equatorial forests. Figshare. (doi:10.6084/m9.figshare.c.8596293)

