# Supplementary material for: Evaluating bromeliads as environmental reservoirs for Batrachochytrium dendrobatidis and amphibian disease dynamics in equatorial forests
Source: R Soc Open Sci. 2026 Sep 9;13(9):rsos252148. doi: 10.1098/rsos.252148 (PMC13561086; doi:10.1098/rsos.252148)
Supplement: Supplementary file 1 [file rsos.252148.zip › rsos.252148/rsos252148_si_001.docx]

**Supplementary Material**

Evaluating bromeliads as environmental reservoirs for *Batrachochytrium dendrobatidis* and amphibian disease dynamics in equatorial forests

MDM Moretta-Urdiales, S. F. McCracken, J. M. Guayasamin, R. L. Lynch, M. Tenorio, W. J. Neely, and D. Rodriguez

Contents:

Supplementary Table 1

Supplementary Table 2

Supplementary Table 3

Supplementary Table 4

Supplementary Table 5

Supplementary Table 6

**Supplementary Table 1.** Pairwise Tukey-adjusted comparisons among amphibian families based on estimated marginal means from the binomial generalized linear model of *Batrachochytrium dendrobatidis* infection status (Bd). The response variable was Bd infection status (0 = negative, 1 = positive), and the model included amphibian families and sampling site as predictors. Reported values include odds ratios, standard errors, z-ratios, and Tukey-adjusted p-values. Odds ratios > 1 indicate greater odds of Bd-positive status in the first family listed in each comparison relative to the second, whereas odds ratios < 1 indicate lower odds. Comparisons are averaged across sampling sites. Because some families were represented by relatively few observations, comparisons should be interpreted cautiously.

| Comparison | Odds ratio | SE | Z ratio | Adjusted p value | Significant |
| --- | --- | --- | --- | --- | --- |
| *Bufonidae / Centrolenidae* | 119044.91 | 50015262.31 | 0.03 | 1.00 | No |
| *Bufonidae / Dendrobatidae* | 0.05 | 0.04 | -3.47 | 0.01 | Yes |
| *Bufonidae / Hylidae* | 0.14 | 0.10 | -2.62 | 0.09 | No |
| *Bufonidae / Leptodactylidae* | 0.08 | 0.06 | -3.21 | 0.02 | Yes |
| *Bufonidae / Strabomantidae* | 0.12 | 0.09 | -2.86 | 0.05 | Yes |
| *Centrolenidae / Dendrobatidae* | 0.00 | 0.00 | -0.03 | 1.00 | No |
| *Centrolenidae / Hylidae* | 0.00 | 0.00 | -0.03 | 1.00 | No |
| *Centrolenidae / Leptodactylidae* | 0.00 | 0.00 | -0.03 | 1.00 | No |
| *Centrolenidae / Strabomantidae* | 0.00 | 0.00 | -0.03 | 1.00 | No |
| *Dendrobatidae / Hylidae* | 2.75 | 1.38 | 2.02 | 0.33 | No |
| *Dendrobatidae / Leptodactylidae* | 1.66 | 0.88 | 0.95 | 0.93 | No |
| *Dendrobatidae / Strabomantidae* | 2.40 | 1.13 | 1.85 | 0.43 | No |
| *Hylidae / Leptodactylidae* | 0.60 | 0.19 | -1.58 | 0.61 | No |
| *Hylidae / Strabomantidae* | 0.87 | 0.19 | -0.62 | 0.99 | No |
| *Leptodactylidae / Strabomantidae* | 1.45 | 0.41 | 1.30 | 0.79 | No |

**Supplementary Table 2.** Pairwise Tukey-adjusted comparisons among amphibian genera based on estimated marginal means from the binomial generalized linear model of *Batrachochytrium dendrobatidis* infection status (Bd). The response variable was Bd infection status (0 = negative, 1 = positive), and the model included amphibian genus and sampling site as predictors. Reported values include odds ratios, standard errors, z-ratios, and Tukey-adjusted p-values. Odds ratios > 1 indicate greater odds of Bd-positive status in the first genus listed in each contrast relative to the second, whereas odds ratios < 1 indicate lower odds. Comparisons are averaged across sampling sites. Because some genera were represented by relatively few observations, comparisons should be interpreted cautiously.

| Comparison | Odds ratio | SE | Z ratio | Adjusted p value | Significant |
| --- | --- | --- | --- | --- | --- |
| *Ameerega / Barycholos* | 4.69 | 5.87 | 1.24 | 1.00 | No |
| *Ameerega / Boana* | 1.59 | 1.16 | 0.63 | 1.00 | No |
| *Ameerega / Dendropsophus* | 0.50 | 0.44 | -0.79 | 1.00 | No |
| *Ameerega / Engystomops* | 0.83 | 0.63 | -0.25 | 1.00 | No |
| *Ameerega / Espadarana* | 4216011.12 | 2920385646.56 | 0.02 | 1.00 | No |
| *Ameerega / Hyloscirtus* | 3.40 | 3.62 | 1.15 | 1.00 | No |
| *Ameerega / Hyloxalus* | 0.52 | 0.49 | -0.69 | 1.00 | No |
| *Ameerega / Leptodactylus* | 1.54 | 1.24 | 0.54 | 1.00 | No |
| *Ameerega / Osteocephalus* | 6.13 | 7.32 | 1.52 | 0.97 | No |
| *Ameerega / Phyllomedusa* | 15.24 | 19.14 | 2.17 | 0.69 | No |
| *Ameerega / Pristimantis* | 1.54 | 1.07 | 0.62 | 1.00 | No |
| *Ameerega / Rhinella* | 13.01 | 12.82 | 2.60 | 0.37 | No |
| *Ameerega / Scinax* | 0.47 | 0.43 | -0.84 | 1.00 | No |
| *Ameerega / Smilisca* | 4755982.14 | 3428770888.82 | 0.02 | 1.00 | No |
| *Barycholos / Boana* | 0.34 | 0.37 | -1.00 | 1.00 | No |
| *Barycholos / Dendropsophus* | 0.11 | 0.13 | -1.89 | 0.86 | No |
| *Barycholos / Engystomops* | 0.18 | 0.20 | -1.57 | 0.97 | No |
| *Barycholos / Espadarana* | 898274.45 | 622225420.25 | 0.02 | 1.00 | No |
| *Barycholos / Hyloscirtus* | 0.73 | 0.95 | -0.24 | 1.00 | No |
| *Barycholos / Hyloxalus* | 0.11 | 0.13 | -1.83 | 0.89 | No |
| *Barycholos / Leptodactylus* | 0.33 | 0.37 | -0.99 | 1.00 | No |
| *Barycholos / Osteocephalus* | 1.31 | 1.97 | 0.18 | 1.00 | No |
| *Barycholos / Phyllomedusa* | 3.25 | 4.81 | 0.79 | 1.00 | No |
| *Barycholos / Pristimantis* | 0.33 | 0.34 | -1.07 | 1.00 | No |
| *Barycholos / Rhinella* | 2.77 | 3.54 | 0.80 | 1.00 | No |
| *Barycholos / Scinax* | 0.10 | 0.12 | -1.95 | 0.83 | No |
| *Barycholos / Smilisca* | 1013322.10 | 730543367.14 | 0.02 | 1.00 | No |
| *Boana / Dendropsophus* | 0.31 | 0.19 | -1.91 | 0.85 | No |
| *Boana / Engystomops* | 0.52 | 0.23 | -1.45 | 0.98 | No |
| *Boana / Espadarana* | 2659257.68 | 1842038487.78 | 0.02 | 1.00 | No |
| *Boana / Hyloscirtus* | 2.15 | 1.84 | 0.89 | 1.00 | No |
| *Boana / Hyloxalus* | 0.33 | 0.23 | -1.59 | 0.96 | No |
| *Boana / Leptodactylus* | 0.97 | 0.49 | -0.05 | 1.00 | No |
| *Boana / Osteocephalus* | 3.87 | 4.31 | 1.21 | 1.00 | No |
| *Boana / Phyllomedusa* | 9.61 | 10.38 | 2.10 | 0.74 | No |
| *Boana / Pristimantis* | 0.97 | 0.28 | -0.10 | 1.00 | No |
| *Boana / Rhinella* | 8.20 | 6.37 | 2.71 | 0.30 | No |
| *Boana / Scinax* | 0.29 | 0.19 | -1.88 | 0.86 | No |
| *Boana / Smilisca* | 2999845.51 | 2162703439.00 | 0.02 | 1.00 | No |
| *Dendropsophus / Engystomops* | 1.66 | 1.08 | 0.77 | 1.00 | No |
| *Dendropsophus / Espadarana* | 8458674.29 | 5859231868.67 | 0.02 | 1.00 | No |
| *Dendropsophus / Hyloscirtus* | 6.83 | 6.71 | 1.96 | 0.82 | No |
| *Dendropsophus / Hyloxalus* | 1.05 | 0.89 | 0.06 | 1.00 | No |
| *Dendropsophus / Leptodactylus* | 3.10 | 2.17 | 1.61 | 0.96 | No |
| *Dendropsophus / Osteocephalus* | 12.31 | 14.99 | 2.06 | 0.76 | No |
| *Dendropsophus / Phyllomedusa* | 30.57 | 35.98 | 2.91 | 0.19 | No |
| *Dendropsophus / Pristimantis* | 3.09 | 1.74 | 2.01 | 0.80 | No |
| *Dendropsophus / Rhinella* | 26.09 | 23.82 | 3.57 | 0.03 | Yes |
| *Dendropsophus / Scinax* | 0.94 | 0.76 | -0.08 | 1.00 | No |
| *Dendropsophus / Smilisca* | 9542029.81 | 6879216028.60 | 0.02 | 1.00 | No |
| *Engystomops / Espadarana* | 5107572.36 | 3537959309.11 | 0.02 | 1.00 | No |
| *Engystomops / Hyloscirtus* | 4.12 | 3.68 | 1.59 | 0.96 | No |
| *Engystomops / Hyloxalus* | 0.63 | 0.47 | -0.62 | 1.00 | No |
| *Engystomops / Leptodactylus* | 1.87 | 1.08 | 1.09 | 1.00 | No |
| *Engystomops / Osteocephalus* | 7.43 | 8.43 | 1.77 | 0.91 | No |
| *Engystomops / Phyllomedusa* | 18.46 | 20.18 | 2.67 | 0.32 | No |
| *Engystomops / Pristimantis* | 1.87 | 0.71 | 1.63 | 0.95 | No |
| *Engystomops / Rhinella* | 15.76 | 12.75 | 3.41 | 0.05 | Yes |
| *Engystomops / Scinax* | 0.57 | 0.39 | -0.82 | 1.00 | No |
| *Engystomops / Smilisca* | 5761731.21 | 4153852838.98 | 0.02 | 1.00 | No |
| *Espadarana / Hyloscirtus* | 0.00 | 0.00 | -0.02 | 1.00 | No |
| *Espadarana / Hyloxalus* | 0.00 | 0.00 | -0.02 | 1.00 | No |
| *Espadarana / Leptodactylus* | 0.00 | 0.00 | -0.02 | 1.00 | No |
| *Espadarana / Osteocephalus* | 1.45E-06 | 0.001008 | -0.0194 | 1.00 | No |
| *Espadarana / Phyllomedusa* | 3.61E-06 | 0.002504 | -0.01809 | 1.00 | No |
| *Espadarana / Pristimantis* | 3.65E-07 | 0.000253 | -0.0214 | 1.00 | No |
| *Espadarana / Rhinella* | 3.08E-06 | 0.002137 | -0.01832 | 1.00 | No |
| *Espadarana / Scinax* | 1.11E-07 | 7.67E-05 | -0.02312 | 1.00 | No |
| *Espadarana / Smilisca* | 1.128076 | 1127.834 | 0.000121 | 1.00 | No |
| *Hyloscirtus / Hyloxalus* | 0.153464 | 0.15758 | -1.82534 | 0.89 | No |
| *Hyloscirtus / Leptodactylus* | 0.45361 | 0.416015 | -0.86196 | 1.00 | No |
| *Hyloscirtus / Osteocephalus* | 1.801867 | 2.438236 | 0.435143 | 1.00 | No |
| *Hyloscirtus / Phyllomedusa* | 4.476522 | 5.93676 | 1.130182 | 1.00 | No |
| *Hyloscirtus / Pristimantis* | 0.452633 | 0.364415 | -0.98456 | 1.00 | No |
| *Hyloscirtus / Rhinella* | 3.820703 | 4.168135 | 1.228703 | 1.00 | No |
| *Hyloscirtus / Scinax* | 0.137157 | 0.137256 | -1.98519 | 0.81 | No |
| *Hyloscirtus / Smilisca* | 1397231 | 1.01E+09 | 0.019627 | 1.00 | No |
| *Hyloxalus / Leptodactylus* | 2.955803 | 2.26873 | 1.411985 | 0.99 | No |
| *Hyloxalus / Osteocephalus* | 11.74128 | 14.79687 | 1.954473 | 0.82 | No |
| *Hyloxalus / Phyllomedusa* | 29.1698 | 35.91397 | 2.739704 | 0.28 | No |
| *Hyloxalus / Pristimantis* | 2.949438 | 1.88019 | 1.696719 | 0.93 | No |
| *Hyloxalus / Rhinella* | 24.89636 | 24.20235 | 3.306904 | 0.06 | No |
| *Hyloxalus / Scinax* | 0.893736 | 0.758497 | -0.13238 | 1.00 | No |
| *Hyloxalus / Smilisca* | 9104603 | 6.56E+09 | 0.022227 | 1.00 | No |
| *Leptodactylus / Osteocephalus* | 3.97228 | 4.622054 | 1.185431 | 1.00 | No |
| *Leptodactylus / Phyllomedusa* | 9.868652 | 11.24619 | 2.008942 | 0.79 | No |
| *Leptodactylus / Pristimantis* | 0.997846 | 0.443378 | -0.00485 | 1.00 | No |
| *Leptodactylus / Rhinella* | 8.422875 | 7.167328 | 2.504243 | 0.44 | No |
| *Leptodactylus / Scinax* | 0.302366 | 0.220687 | -1.63881 | 0.95 | No |
| *Leptodactylus / Smilisca* | 3080246 | 2.22E+09 | 0.020724 | 1.00 | No |
| *Osteocephalus / Phyllomedusa* | 2.48438 | 3.745323 | 0.603644 | 1.00 | No |
| *Osteocephalus / Pristimantis* | 0.251202 | 0.273293 | -1.26983 | 1.00 | No |
| *Osteocephalus / Rhinella* | 2.120413 | 2.745281 | 0.580533 | 1.00 | No |
| *Osteocephalus / Scinax* | 0.076119 | 0.094156 | -2.08209 | 0.75 | No |
| *Osteocephalus / Smilisca* | 775435.3 | 5.59E+08 | 0.01881 | 1.00 | No |
| *Phyllomedusa / Pristimantis* | 0.101113 | 0.106584 | -2.17389 | 0.68 | No |
| *Phyllomedusa / Rhinella* | 0.853498 | 1.086875 | -0.1244 | 1.00 | No |
| *Phyllomedusa / Scinax* | 0.030639 | 0.036869 | -2.8965 | 0.20 | No |
| *Phyllomedusa / Smilisca* | 312124.3 | 2.25E+08 | 0.017548 | 1.00 | No |
| *Pristimantis / Rhinella* | 8.441054 | 6.233516 | 2.888526 | 0.20 | No |
| *Pristimantis / Scinax* | 0.303019 | 0.180202 | -2.00771 | 0.79 | No |
| *Pristimantis / Smilisca* | 3086895 | 2.23E+09 | 0.020727 | 1.00 | No |
| *Rhinella / Scinax* | 0.035898 | 0.033795 | -3.53413 | 0.03 | Yes |
| *Rhinella / Smilisca* | 365700.1 | 2.64E+08 | 0.017768 | 1.00 | No |
| *Scinax / Smilisca* | 10187132 | 7.34E+09 | 0.022383 | 1.00 | No |

**Supplementary Table 3.** *Batrachochytrium dendrobatidis* (Bd) prevalence across sampling sites in Ecuador for amphibian hosts and bromeliads. Bromeliad samples consist of eDNA from water and soil collected from each tank. This table includes site-specific prevalence estimates with 95% confidence intervals, sample sizes (N hosts), and amphibian diversity metrics, including: number of host families (N. host families) and genera (N. host genera). These values provide a comparison of Bd infection between amphibians and bromeliads at each site.

| Site | Bd Prevalence | Source | N. hosts | N. host families | N. host genera |
| --- | --- | --- | --- | --- | --- |
| Cosanga | 8% (2.2 - 19.2) | frog | 50 | 4 | 5 |
| JCR | 14.9% (11.1 - 19.4) | frog | 302 | 6 | 12 |
| Lalo Loor | 11.8% (4.4 - 23.9) | frog | 51 | 4 | 5 |
| Mashpi | 34% (27.6 - 40.8) | frog | 209 | 7 | 8 |
| Saloya | 20.2% (15.2 - 25.9) | frog | 233 | 4 | 7 |
| Tandayapa | 12.7% (7.3 - 20.1) | frog | 118 | 1 | 1 |
| Tiputini | 18.4% (11.3 - 27.5) | frog | 98 | 7 | 11 |
| Zanja Arajuno | 42.1% (35.2 - 49.2) | frog | 202 | 6 | 17 |
| Cosanga | 16.7% (8.3 - 28.5) | bromeliads | 60 | - | - |
| JCR | 0% (0 - 6) | bromeliads | 60 | - | - |
| Mashpi | 11.7% (4.8 - 22.6) | bromeliads | 60 | - | - |
| Saloya | 3.3% (0.4 - 11.5) | bromeliads | 60 | - | - |
| Tandayapa | 8.3% (2.8 - 18.4) | bromeliads | 60 | - | - |
| Tiputini | 13.3% (5.9 - 24.6) | bromeliads | 60 | - | - |
| Zanja Arajuno | 15% (7.1 - 26.6) | bromeliads | 60 | - | - |

**Supplementary Table 4.** *Batrachochytrium dendrobatidis* (Bd) prevalence across amphibian genera sampled in Ecuador over a three-year period. For each genus, the table reports Bd prevalence, exact 95% confidence intervals, total sample size (N), and number of Bd-positive individuals (N. infected).

| Genus | Bd prevalence (95% CI) | N | N. infected |
| --- | --- | --- | --- |
| *Pristimantis* | 22.9% (20.1 - 25.8) | 870 | 199 |
| *Boana* | 26.6% (17.3 - 37.7) | 79 | 21 |
| *Engystomops* | 55.6% (38.1 - 72.1) | 36 | 20 |
| *Dendropsophus* | 60% (32.3 - 83.7) | 15 | 9 |
| *Leptodactylus* | 20% (8.4 - 36.9) | 35 | 7 |
| *Scinax* | 46.2% (19.2 - 74.9) | 13 | 6 |
| *Adenomera* | 66.7% (22.3 - 95.7) | 6 | 4 |
| *Ameerega* | 26.7% (7.8 - 55.1) | 15 | 4 |
| *Hyloxalus* | 33.3% (9.9 - 65.1) | 12 | 4 |
| *Epipedobates* | 33.3% (7.5 - 70.1) | 9 | 3 |
| *Hyloscirtus* | 20% (2.5 - 55.6) | 10 | 2 |
| *Oreobates* | 25% (3.2 - 65.1) | 8 | 2 |
| *Rhinella* | 3.7% (0.5 - 12.7) | 54 | 2 |
| *Allobates* | 50% (1.3 - 98.7) | 2 | 1 |
| *Barycholos* | 5.3% (0.1 - 26) | 19 | 1 |
| *Craugastor* | 20% (0.5 - 71.6) | 5 | 1 |
| *Osteocephalus* | 6.2% (0.2 - 30.2) | 16 | 1 |
| *Phyllomedusa* | 7.1% (0.2 - 33.9) | 14 | 1 |
| *Sachatamia* | 33.3% (0.8 - 90.6) | 3 | 1 |
| *Teratohyla* | 100% (2.5 - 100) | 1 | 1 |
| *Trachycephalus* | 25% (0.6 - 80.6) | 4 | 1 |
| *Agalychnis* | 0% (0 - 97.5) | 1 | 0 |
| *Centrolene* | 0% (0 - 97.5) | 1 | 0 |
| *Chiasmocleis* | 0% (0 - 97.5) | 1 | 0 |
| *Espadarana* | 0% (0 - 26.5) | 12 | 0 |
| *Hyalinobatrachium* | 0% (0 - 45.9) | 6 | 0 |
| *Lithodytes* | 0% (0 - 97.5) | 1 | 0 |
| *Nymphargus* | 0% (0 - 84.2) | 2 | 0 |
| *Rulyrana* | 0% (0 - 84.2) | 2 | 0 |
| *Smilisca* | 0% (0 - 28.5) | 11 | 0 |

**Supplementary Table 5.** *Batrachochytrium dendrobatidis* (Bd) prevalence across amphibian families represented in the analytical dataset used for family-level models. For each family, the table reports Bd prevalence, exact 95% confidence intervals, total sample size (N), and number of Bd-positive individuals (N. infected).

| Family | Bd prevalence (95% CI) | N | N. infected |
| --- | --- | --- | --- |
| Strabomantidae | 22.5% (19.8 - 25.4) | 897 | 202 |
| Hylidae | 25.2% (18.7 - 32.5) | 163 | 41 |
| Leptodactylidae | 39.7% (28.8 - 51.5) | 78 | 31 |
| Dendrobatidae | 30.6% (16.3 - 48.1) | 36 | 11 |
| Bufonidae | 3.7% (0.5 - 12.7) | 54 | 2 |
| Centrolenidae | 7.4% (0.9 - 24.3) | 27 | 2 |
| Aromobatidae | 50% (1.3 - 98.7) | 2 | 1 |
| Craugastoridae | 20% (0.5 - 71.6) | 5 | 1 |
| Microhylidae | 0% (0 - 97.5) | 1 | 0 |

**Supplementary Table 6.** Environmental conditions at sampling sites across Ecuador. The table reports mean air temperature and standard deviation (± SD), precipitation (measured in millimeters), month, and year of sampling. These climatic parameters provide context for interpreting variation in *Batrachochytrium dendrobatidis* (Bd) infection across sites and environmental conditions.

| Site | Mean Temperature (°C) | Mean Precipitation (mm) | Month of Sampling | Year of Sampling |
| --- | --- | --- | --- | --- |
| Tandayapa | 14.6 ± 1.4 | 91.1 | December | 2022 |
| Cosanga | 12.3 ± 2.2 | 183 | June | 2023 |
| JCR | 23.6 ± 1.6 | 26.9 | June | 2021 |
| JCR | 23.2 ± 1.6 | 66.4 | December | 2021 |
| JCR | 22.9± 1.5 | 46.9 | June | 2022 |
| JCR | 22.8 ± 1.8 | 16.4 | July | 2022 |
| Mashpi | 20.5 ± 1.5 | 190.5 | July | 2021 |
| Saloya | 17.1 ± 1.3 | 77.3 | July | 2022 |
| Saloya | 17.0 ± 1.3 | 273.7 | January | 2023 |
| Tiputini | 24 ± 2.3 | 483 | June | 2023 |
| Zanja Arajuno | 22 ± 2.1 | 363.3 | December | 2023 |
| Lalo Loor | 23.2 ± 1.3 | 54.1 | June | 2022 |
